# The Terpene Mini-Path, a New Promising Alternative for Terpenoids Bio-Production

**DOI:** 10.3390/genes12121974

**Published:** 2021-12-10

**Authors:** Julie Couillaud, Létitia Leydet, Katia Duquesne, Gilles Iacazio

**Affiliations:** 1 Centrale Marseille, CNRS, iSm2 Marseille, ISM2 UMR 7313, Aix-Marseille Université, Av. Escadrille Normandie-Niemen, 13013 Marseille, France; julie.couillaud.13990@gmail.com (J.C.); letitia.LEYDET@univ-amu.fr (L.L.); katia.duquesne@univ-amu.fr (K.D.); 2 Systems and Synthetic Biology Division, Department of Biology and Biological Engineering, Chalmers University of Technology, 41296 Gothenburg, Sweden

**Keywords:** terpenoids bio-access, terpene mini-path, synthetic biology

## Abstract

Terpenoids constitute the largest class of natural compounds and are extremely valuable from an economic point of view due to their extended physicochemical properties and biological activities. Due to recent environmental concerns, terpene extraction from natural sources is no longer considered as a viable option, and neither is the chemical synthesis to access such chemicals due to their sophisticated structural characteristics. An alternative to produce terpenoids is the use of biotechnological tools involving, for example, the construction of enzymatic cascades (cell-free synthesis) or a microbial bio-production thanks to metabolic engineering techniques. Despite outstanding successes, these approaches have been hampered by the length of the two natural biosynthetic routes (the mevalonate and the methyl erythritol phosphate pathways), leading to dimethylallyl diphosphate (DMAPP) and isopentenyl diphosphate (IPP), the two common universal precursors of all terpenoids. Recently, we, and others, developed what we called the terpene mini-path, a robust two enzyme access to DMAPP and IPP starting from their corresponding two alcohols, dimethylallyl alcohol and isopentenol. The aim here is to present the potential of this artificial bio-access to terpenoids, either in vitro or in vivo, through a review of the publications appearing since 2016 on this very new and fascinating field of investigation.

## 1. Introduction

**Scheme 1:**
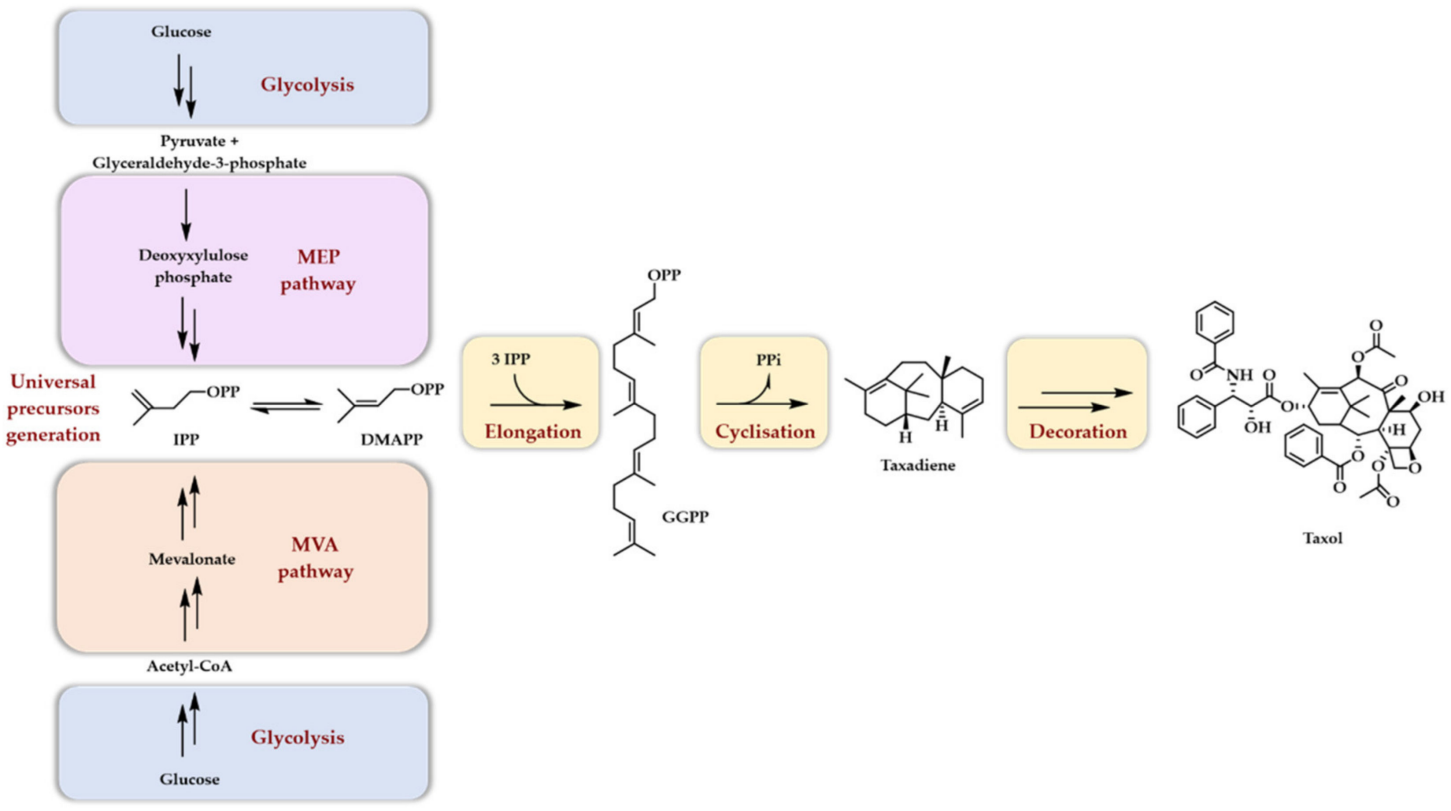
Simplified biosynthetic scheme of terpene biosynthesis with a special focus put on taxol biosynthesis. It should be noted that, in plants, diterpenoids (such as taxol) arise mainly from the plastidial MEP pathway.

Taxol, artemisinin, menthol, camphor, cholesterol, beta-carotene, and rubber are just a few very well-known names of terpenoids of some interest for humanity. As a natural product family, terpenoids have long attracted scientific interest due to their very diversified physical and chemical properties as well as their numerous biological activities. Another point of great interest lies in their unique biosynthetic scheme. Indeed, two mechanisms, called the mevalonate pathway (MVA) and the methyl erythritol phosphate (MEP) pathway together with glycolysis, convert glucose into the two five carbon atoms, universal precursors of terpenoids: dimethylallyl diphosphate (DMAPP) and isopentenyl diphosphate (IPP). Both pathways involve an 18 enzymes cascade. The MVA pathway is mainly found in eukaryotes and archaebacteria, whereas the MEP pathway is mainly found in eubacteria and chloroplasts. Once synthesized, DMAPP and IPP are condensed together to generate, in an extension process, linear diphosphates of different lengths characterized by a carbon number multiple of five. The extraordinary structural diversity of terpenoids comes next. Indeed, these linear diphosphates are first cyclized by numerous terpene synthases/cyclases, generating cyclic hydrocarbons but also alcohols or ethers (see refs [1,2,3,4,5,6] for a detailed view of their mechanism of action), giving rise to monoterpenes (C10), sesquiterpenes (C15), diterpenes (C20), sesterterpenes (C25), triterpenes (C30), tetraterpenes (C40), and higher homologs depending on the linear diphosphate used as a starter unit (DMAPP, geranyl diphosphate GPP, farnesyl diphosphate FPP, geranylgeranyl diphosphate GGPP and the number of extension units added (IPP). A second layer of diversity is then added when the initially formed scaffold is enzymatically decorated with a myriad of oxidases (cytochrome P450, α-ketoglutarate dependent dioxygenases), glyco- and acyl-transferases, as well as dehydrogenases and halogenases. These two steps of modification, cyclisation and decoration, are, thus, the basis of terpenoids’ structural diversity. The first step usually involves a single enzyme, while the second, depending on the final product structure, may involve more than ten enzymes. As a projecting example, we provide here a simplified biosynthesis scheme of taxol (Scheme 1), the whole sequence, particularly during the decoration phase, being actually not entirely known [7].

It should also be noted that DMAPP, as well as diphosphates of higher length, such as GPP, FPP, and GGPP, can be coupled to natural molecules of different biosynthetic origins, such as amino acids or polyphenols, and further processed, offering another source of structural diversity.

**Scheme 2:**
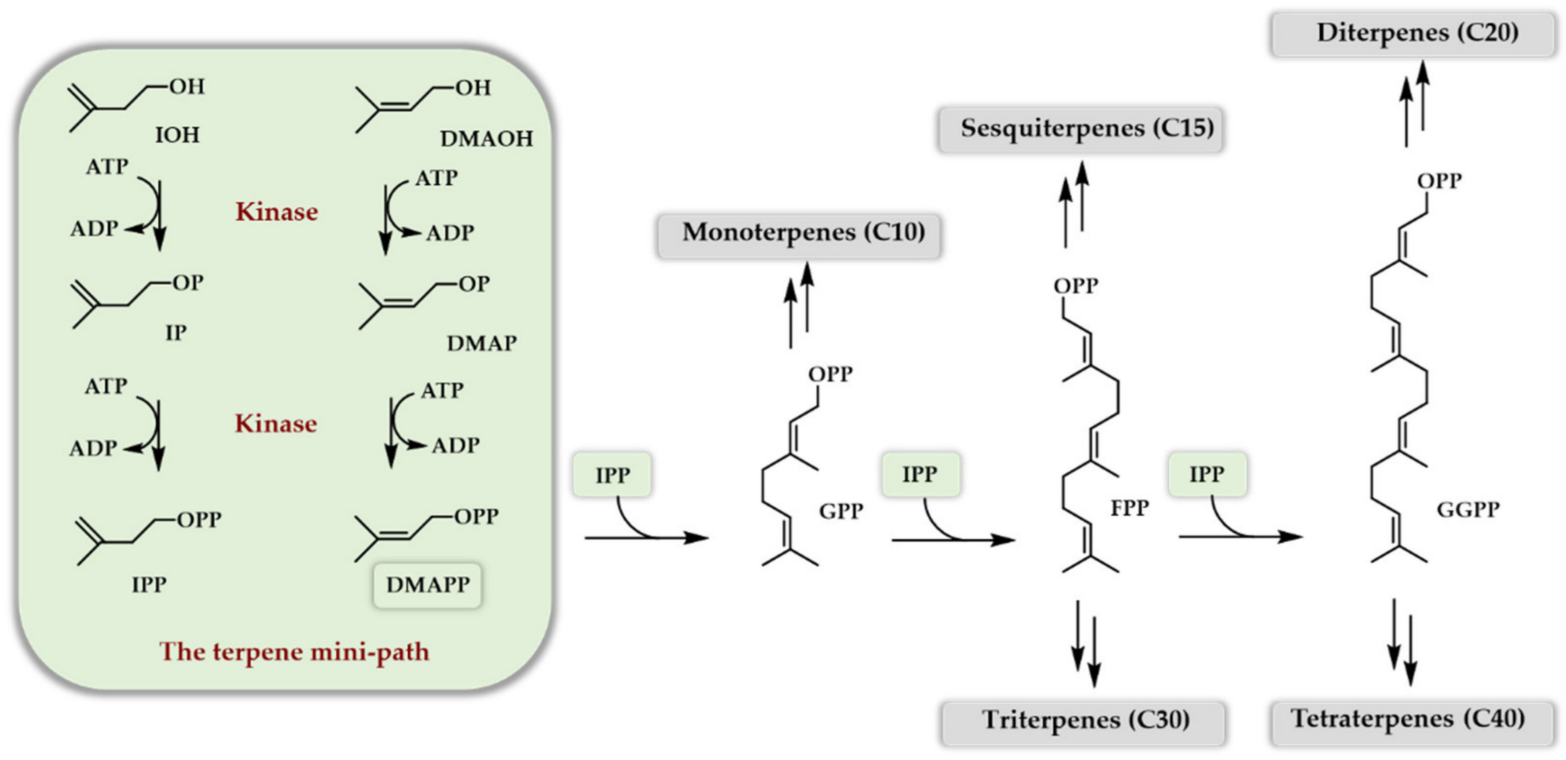
Simplified enzymatic access to DMAPP and IPP, as well as terpenoids, through the TMP.

In the first decade of this century, a masterpiece of work was engaged by the Keasling lab in order to produce artemisinin, a sequiterpenoid with antimalarial activity found in the plant *Artemisia annua*. Impressive titers of amorphadiene (the sesquiterpene hydrocarbon precursor of artemisinin, 40 g/L) and of artemisinic acid (the starting synthon for the chemical access to artemisinin, 25 g/L) were obtained in cultures of recombinant yeasts bearing an optimized MVA pathway [8] at the expense of 150 persons per year of work [9]. One of the difficulties of this approach is that the carbon and energy source of the recombinant microbe is also the biosynthetic source of carbon of the terpenoid of interest, which makes balancing the flux towards the latter quite delicate. Having a look at the biosynthetic scheme of all the terpenes (Scheme 1), it is of note that the road to DMAPP and IPP starting from glucose is not the simplest in terms of chemical modifications. Indeed, not from a biological perspective but from a synthetic chemistry point of view, it is nonsense to start from glucose to access DMAPP or IPP. At this point of reflection, the simplest way to access DMAPP and IPP with minimal structural modifications is to use dimethylallyl alcohol (DMAOH) and isopentenol (IOH), the two homologous alcohols of DMAPP and IPP, and perform a double phosphorylation. If a one- or two-step enzymatic phosphorylation access to DMAPP and IPP becomes available starting from DMAOH and IOH, then the paradigm of terpenoids’ bio-production could be changed and give rise to a new and easy way to the industrial access of those outstanding chemicals. Indeed, if successful, the implementation of this new artificial pathway offers the possibility to decouple microbial growth (use of glucose or glycerol as carbon and energy sources) and terpenoid production (use of DMAOH and IOH as biosynthetic carbon), hence simplifying the process of optimization. In addition, the use of only one or two enzyme(s) to doubly phosphorylate DMAOH and IOH makes this artificial route usable both in vivo and in vitro due to its extreme simplicity. Best of all, DMAOH and IOH are both readily available bulk chemicals and can potentially be bio-sourced. The idea became true when four groups reported independently in 2019 [10,11,12,13] the capacity of a two enzymes cascade to generate DMAPP and IPP from DMAOH and IOH in order to produce, either in vitro or in vivo, various terpenoids. These findings followed soon the initial demonstration that only one enzyme could also manage the double phosphorylation of both DMAOH and IOH [14,15] but with a low yield. In the following, we will review the development of this new way of producing terpenoids, which we called the terpene mini-path (TMP), using only one or two enzymes either in vitro (purified enzymes, cell-free systems) or in vivo (microbial fermentations) to access DMAPP and IPP, and we will discuss the potential applications of this new artificial bio-access to terpenoids (Scheme 2).

## 2. Premises

The TMP is *a priori* totally artificial. Indeed, DMAOH and IOH are not very common chemicals found in living organisms and, if generated, they are considered to occur from DMAPP and IPP by (di)phosphatase action rather than the opposite. Nevertheless, some experimental evidence has been provided that DMAOH and IOH can be used either in a mixture or individually by some microorganisms to obtain higher titers in terpenoids when exogenously added to the culture medium. It should be noted that no enzymes were described nor suggested to be responsible for the incorporation of the two alcohols in these initial studies. Using a bacterium, different yeasts, and a filamentous fungus, Kawada et al. [16] demonstrated that the addition of DMAOH, IOH, geraniol (GOH), or mixtures of IOH with either DMAOH or GOH at concentrations of 0.5 mM–1 mM, improves the ubiquinone content of the considered cells by 30 to 72% at the end of the fermentation process. The same was also demonstrated later on using *Rhizobium radiobacter* [17] with a cellular increase of 30% in ubiquinone content following the addition in the culture medium of 1 mM IOH. In a completely different type of experiment aiming at the time to discover the late enzymes (genes) of the MEP pathway, it was demonstrated that an insertion in the promotor region of the *lytB* gene of *Synechocystis* strain PCC 6803 caused impaired growth and a greenish-yellow phenotype instead of the dark blue-green normal one. The latter was recovered, as well as the growth capacity when DMAOH and IOH were supplemented to the solid culture media [18]. Slightly later, the LytB enzyme was proved to be the last enzyme of the MEP pathway catalyzing the transformation of 1-hydroxy-2-methyl-2-(*E*)-butenyl 4-diphosphate into a mixture of IPP and DMAPP with a 5:1 ratio [19]. In the three reported cases, the role of added DMAOH and IOH in increasing ubiquinone production or in restoring the normal phenotype can be explained by their transformation into DMAPP or IPP by endogenous kinases as proposed earlier [18]. This suggests the existence of a potential natural capacity to produce terpenoids from DMAOH and IOH. Nevertheless, in the absence of a specific biosynthetic route to access DMAOH and IOH, except from DMAPP and IPP by action of (di)phosphatases, the capacity to use both C5 alcohols to generate terpenoids is probably metabolically irrelevant or could be a futile cycle in the worst case or a DMAPP/IPP endogenous salvage pathway in the best one (see below). When put together, all these experiments point to the existence of a natural enzyme(s) able to doubly phosphorylate either one or both of the C5 alcohols, DMAOH and IOH.

## 3. One Enzyme Access to DMAPP/IPP

### 3.1. The Isopentenyl Phosphate Kinase Enzyme

**Scheme 3:**
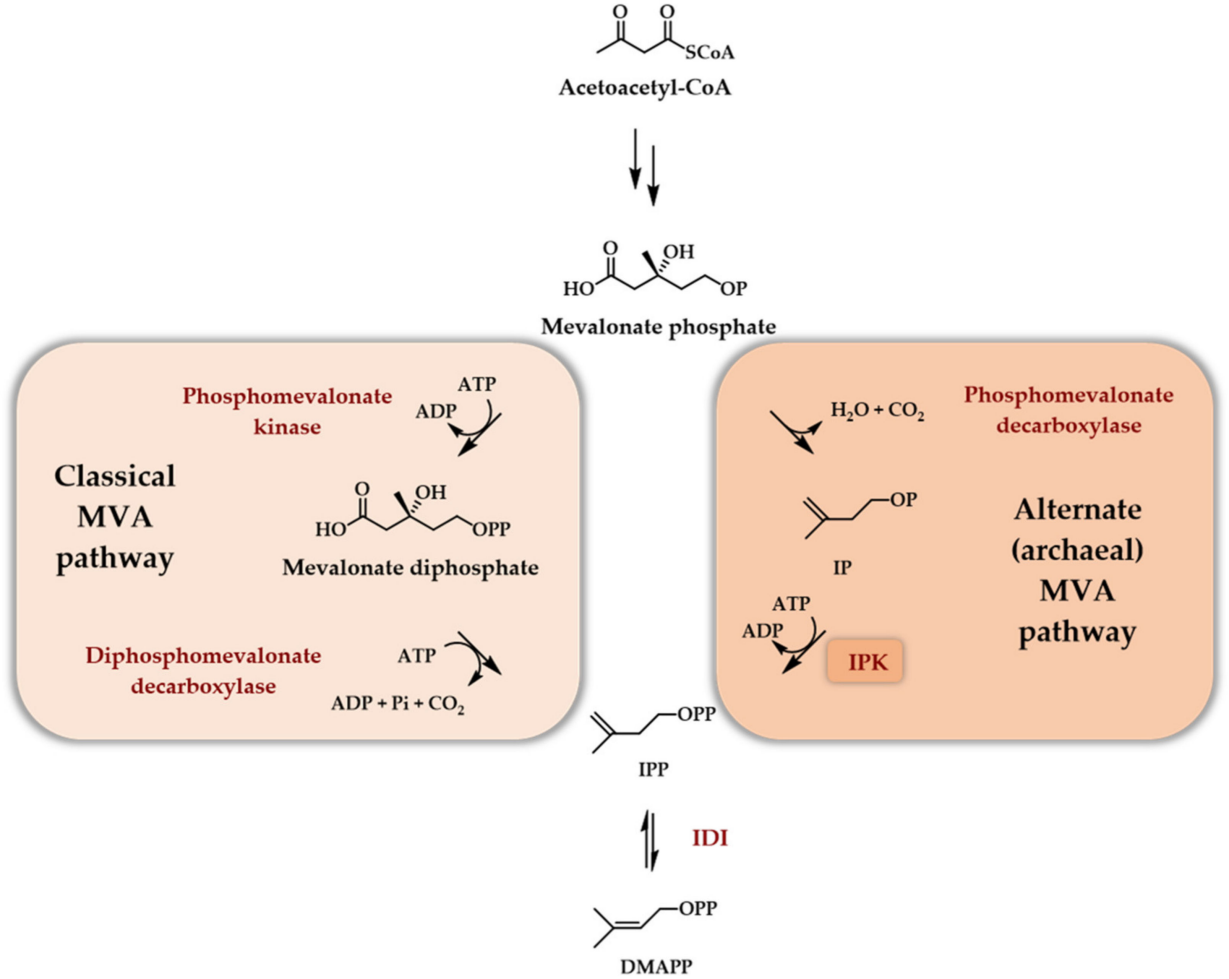
Role of IPK in the alternate archaeal MVA pathway.

Before being recognized as part of a modified mevalonate pathway in archaea [20], isopentenyl phosphate kinase (IPK) was first detected in *E. coli* and in *Mentha piperita* [21] and was believed at the time to be the last enzymatic step of the not-yet-elucidated MEP pathway [21]. Using the recombinant and partially purified *E. coli* IPK (IPK*_Ec_*), it was demonstrated that the best tested enzyme substrate in the presence of ATP was isopentenyl phosphate (IP) generating isopentenyl diphosphate (Scheme 3).

**Scheme 4:**
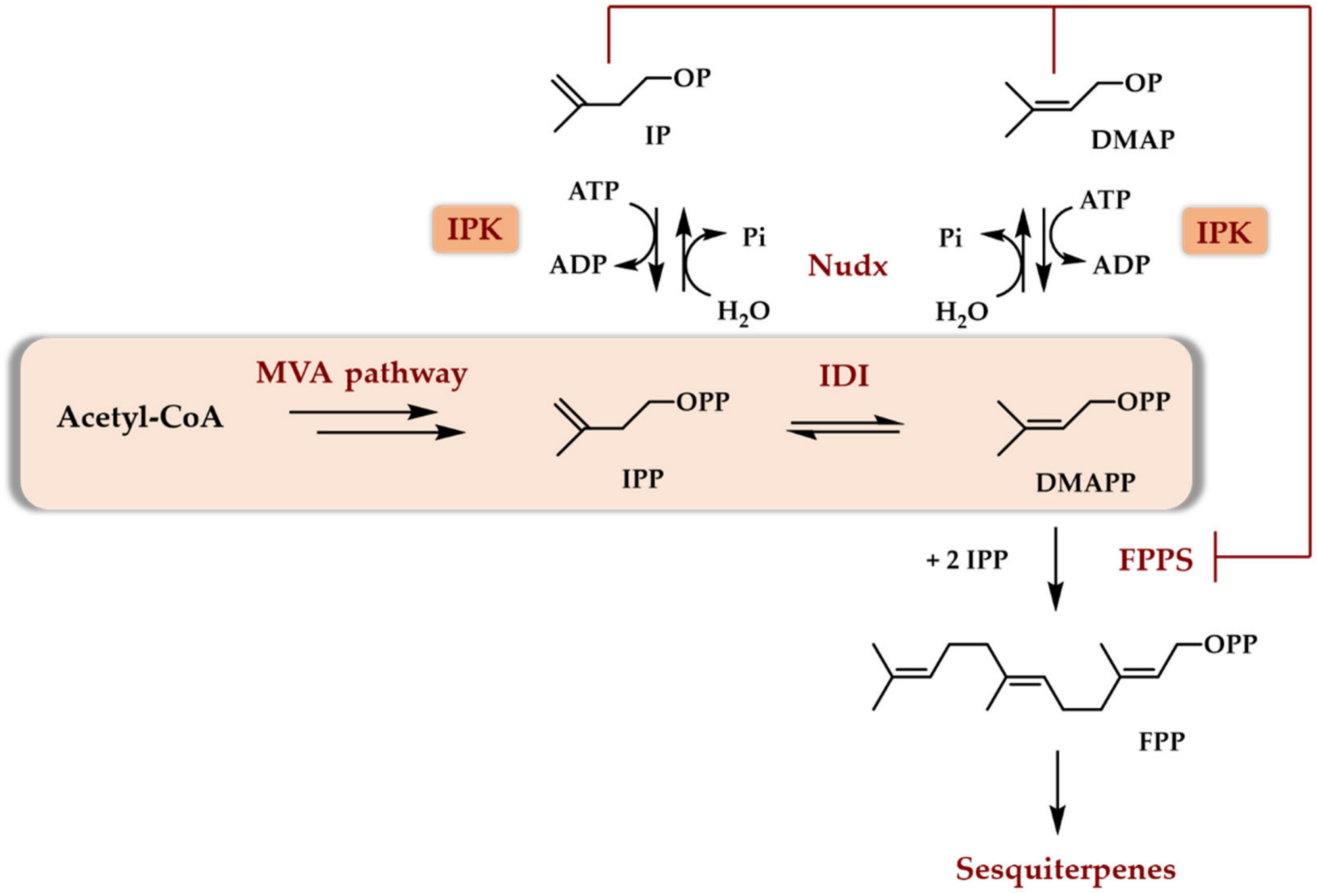
Potential physiological role of IPKs (and of Nudx) in the regulation of sesquiterpenoids biosynthesis in plants. IDI stands for isopentenyl diphosphate isomerase. Nudx stands for Nudx phosphatase. FPPS stands for farnesyl diphosphate synthase (adapted from [25]).

The enzyme proved also to catalyze the phosphorylation of IOH with a non-negligible relative activity of 44% when compared to IP. Low activity towards DMAOH and no activity towards DMAP were noticed [21]. Although not recognized as such at the time, this pioneering work was the first to name a putative enzyme involved in the transformation of IOH to IPP and thus demonstrated the potential of a very short enzymatic access to terpenoids starting from IOH. With the demonstration that the last step in the MEP pathway was independent of IP, no further work, to the best of our knowledge, was devoted to the physiological role of *E. coli* IPK*_Ec_* in the following years. Building on the extensive whole genome sequencing effort done at the beginning of the century, it was quickly recognized that numerous archaebacteria genomes lack the two final genes of the classical MVA pathway, that is the genes coding for phosphomevalonate kinase and diphosphomevalonate decarboxylase (Scheme 3). Using Methanocaldococcus jannaschii and Haloferax volcanii as model organisms, it was demonstrated that these two enzymes are replaced by a phosphomevalonate decarboxylase [22] and an IPK [23] within archaea. Thus, at least in archaea, the physiological role of IPKs was assessed as being part of an alternate MVA pathway (Scheme 3). Later, it was recognized by bioinformatic studies that each sequenced plant genome possessed an IPK encoding gene [24]. It is, therefore, relevant to consider what the physiological role of this enzyme is in plants. Recently, some studies have demonstrated a potential physiological role of plant IPKs in conjunction with hydrolases from the Nudx family, both enzymes being supposed to be involved in the regulation of DMAPP/DMAP and IPP/IP ratios in plant cytosol ([25], reviewed in [24]). Indeed, the ability of DMAP and IP to competitively inhibit farnesyl diphosphate synthase and the demonstrated presence of both IPK and phosphatase acting specifically on DMAPP and IPP but not on DMAP or IP led to a regulatory scheme [25]. This latter describes the central role of the DMAP/IP pair in the regulation of the biosynthesis of terpenoids through the cytosolic MVA pathway, at least in plants (Scheme 4).

Thus, at the beginning of the century, one natural enzyme, IPK, was recognized as widely distributed in the tree of life with different physiological roles and catalyzing the phosphorylation of IP into IPP. This enzyme was also proved to be promiscuous enough to catalyze the transformation of DMAP into DMAPP [26]. In the perspective of building a short enzymatic access to DMAPP/IPP from DMAOH/IOH, the second phosphorylation step looked thus to be secure thanks to the IPK. From the work on the IPK*_Ec_* [21], it was even conceivable to establish a one enzyme double phosphorylation of at least IOH into IPP thanks to IPK.

### 3.2. One Enzyme Double Phosphorylation of DMAOH and IOH

The one enzyme double phosphorylation of DMAOH and IOH is obviously the shortest route to access the universal precursors DMAPP and IPP. Two papers have been devoted to such a restricted cascade, both taking advantage of the ability of IPKs to catalyze this double phosphorylation. In one case, the report is based on protein engineering of IPKs by analysis of amino acid coevolution [14]; the other is focused on screening of IPK biodiversity [15]. Liu et al. were the first to use the ability of IPKs to catalyze, in addition to their normal IP kinase activity leading to IPP, another kinase activity on DMAOH, potentially leading to the construction of a one enzyme two step access to DMAPP [14]. It should be noted that the DMAOH kinase catalytic efficiency of *Thermoplasma acidophilum* IPK (IPK*_Ta_*) was three orders of magnitude lower than the normal IP kinase activity [14,23]. In order to improve the DMAOH kinase activity, the coevolution of the IPK protein sequences was analyzed and potential amino acid positions were detected that can, if mutated, increase the catalytic activity of IPK*_Ta_* towards DMAOH. Single, double, or triple mutants were generated, the best ones showing up to an eight-fold increase in catalytic activity. This protein engineering approach was used to produce in vivo (*E. coli*) β-carotene. A maximum of 3.78 mg of β-carotene per gram of dry cell weight was obtained in the best case, involving a triple mutant of IPK*_Ta_*.

As part of a larger project, various IPKs extracted from biodiversity were tested as potential catalysts to be produced in *E. coli* in a soluble active form, usable at pH 7, 37 °C, and with ATP as a phosphorylating agent. From the 93 over-expressed proteins, 66 were correctly produced after IPTG addition, but only 24 were soluble. Five IPKs exhibiting high isopentenyl phosphate kinase activity with low sequence similarity to IPK*_Ta_* were further chosen and their genes combined individually with the genes encoding the enzymes involved in the generation of lycopene (crtE, crtB, and crtI). When adding DMAOH and IOH to the culture medium, a maximum 11-fold increase in carotenoid content was obtained with the IPK of *Methanococcus maripaludis* as a catalyst. Unexpectedly, it was, furthermore, observed that the main formed carotenoid was neurosporene (up to 94%) at a concentration of 0.7 mg per gram of dry cell weight [15]. In both cases [14,15], the production of carotenoids has been proved to be associated with the expression of IPKs.

These two reports are, to our knowledge, the only ones dealing with a one enzyme TMP to generate in vivo both DMAPP and IPP from DMAOH and IOH. Unfortunately, regarding whether IPKs are efficient enzymes to transform DMAP and IP into DMAPP and IPP, their catalytic efficiency dropped drastically when tested as DMAOH/IOH kinase. As an IPK having equivalent and high activities for both phosphorylation steps is yet to be found or created, various teams used promiscuous enzymes in order to catalyze the first DMAOH/IOH phosphorylation step (no known enzymes being dedicated to this transformation in nature), in combination with an IPK, in order to generate an efficient two enzyme TMP.

## 4. Two Enzyme Access to DMAPP/IPP

### 4.1. What Enzyme to Be Coupled with an IPK

As mentioned earlier, to develop an efficient and short artificial TMP, it is necessary to couple a DMAP/IP kinase to an IPK in order to generate the two C5 corresponding diphosphates. One option, adopted in two of the first reports on the TMP, was to use a phosphatase to perform the first phosphorylation step [11,12]. Indeed, phosphatases catalyze the generally reversible hydrolysis of organic phosphates, leading to an alcohol and a phosphate ion. It was demonstrated that bacterial acidic phosphatases of the PhoN family were able to catalyze the reverse reaction, i.e., the synthesis of monophosphates starting from alcohols, and especially primary ones, using ATP or diphosphate as a phosphorylating agent [27]. Of particular relevance was the fact that such an enzyme was found to be able to catalyze the formation of isopentanyl phosphate from isopentanol, the hydrogenated equivalent of DMAOH and IOH [27]. Obviously, the use of a phosphatase poses the problem of the unwanted reverse hydrolysis of the newly formed monophosphate. However, if the monophosphate is immediately transformed into the corresponding diphosphate, double phosphorylation can be secured, especially if the enzymatic cascade can be pushed forward by coupling with a prenyl transferase consuming the formed diphosphates. In another pioneering work [10], *Saccharomyces cerevisiae* choline kinase was found, when screening potentially useful kinases for DMAOH/IOH phosphorylation, to be the one with the most interesting kinetic parameters. It was subsequently used further to develop the so-called isopentenol utilization pathway (IUP). Very recently, a kinase described as acting on 4-methyl-5-hydroxyethylthiazole (ThiM kinase) was proved to be promiscuous enough to catalyze the phosphorylation of DMAOH into DMAP [28]. The latter is the prenyl donor in the generation of prenylated FMN, a recently discovered cofactor used by the UbiD family of reversible decarboxylases [28]. In *E. coli*, DMAP can be generated either by Nudx phosphatase from DMAPP or by ThiM kinase from DMAOH. The presence of both ThiM and IP kinases in *E. coli* could, by analogy, explain the reported ability of some microorganisms to use added DMAOH and IOH as substrates for the generation of DMAPP/IPP, as shown above. ThiM kinase was also proved to be useful for IOH as a substrate and was, therefore, combined with IPKs to produce the universal diphosphates in vitro [29,30] or in vivo [13] in order to generate various terpenoids using DMAOH/IOH as precursors.

### 4.2. The TMP In Vitro—Purified Enzymes

**Scheme 5:**
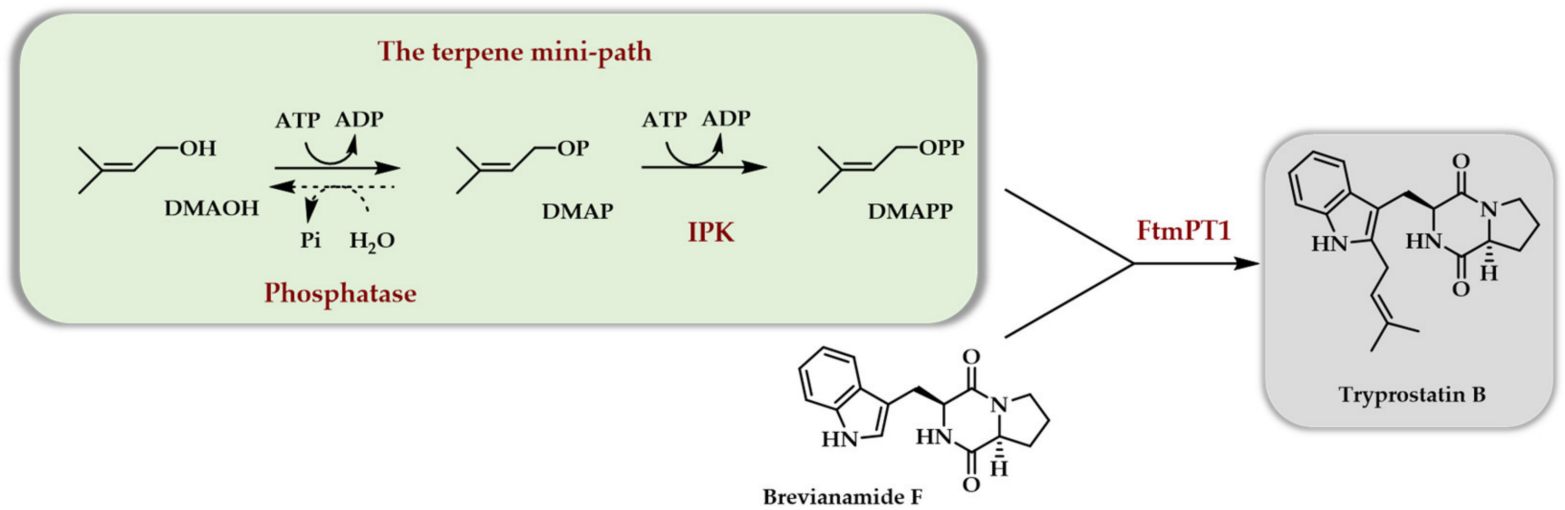
First in vitro use of the TMP to access tryprostatin B in a three enzymes-one pot cascade [12].

The first report dealing with the use of purified enzymes to access terpenoids from C5 alcohols demonstrated that the combination of a phosphatase and an IPK could generate DMAPP starting from DMAOH [12]. As a proof of concept, the former was used as a prenyl donor to generate a cytotoxic prenylated diketopiperazine compound, tryprostatin B, using chemically synthesized brevianamide F (BF) and prenyl transferase FtmPT1 from *Aspergillus fumigatus* as a catalyst (Scheme 5).

A large optimization of the three enzymes cascade allowed to totally transform BF into tryprostatin B in 24 h on a 10 mM scale. The main influencing parameters were found to be the presence of an ATP recycling system, an increase in DMAOH concentration *versus* BF concentration, a double ATP addition during the reaction, a decreased concentration in phosphatase, and a higher concentration in prenyl transferase FtmPT1 as compared to initial conditions. This optimization was made necessary by the use of a phosphatase for the first enzymatic step. Indeed, it was demonstrated that this phosphatase was able to quickly hydrolyze any of the phosphates or diphosphates added in the reaction medium or produced during the reaction (DMAP, DMAPP, PEP, AMP, ADP, ATP). As a general rule, influencing parameters acting either to slow down phosphate and diphosphate hydrolysis or to maintain a high level in phosphorylating agent (low phosphatase activity, ATP regeneration system, addition of ATP) or to promote the production and use of DMAPP (higher DMAOH concentration, higher prenyl transferase activity) had to counteract phosphatase activity. While the proof of concept of the in vitro usefulness of the TMP was completed and a final concentration of tryprostatin B of ~3 g/L was obtained, the TMP using a phosphatase as the first enzyme did not appear to be the best option. It should be noted that the used enzymes were freshly purified and not frozen.

**Scheme 6:**
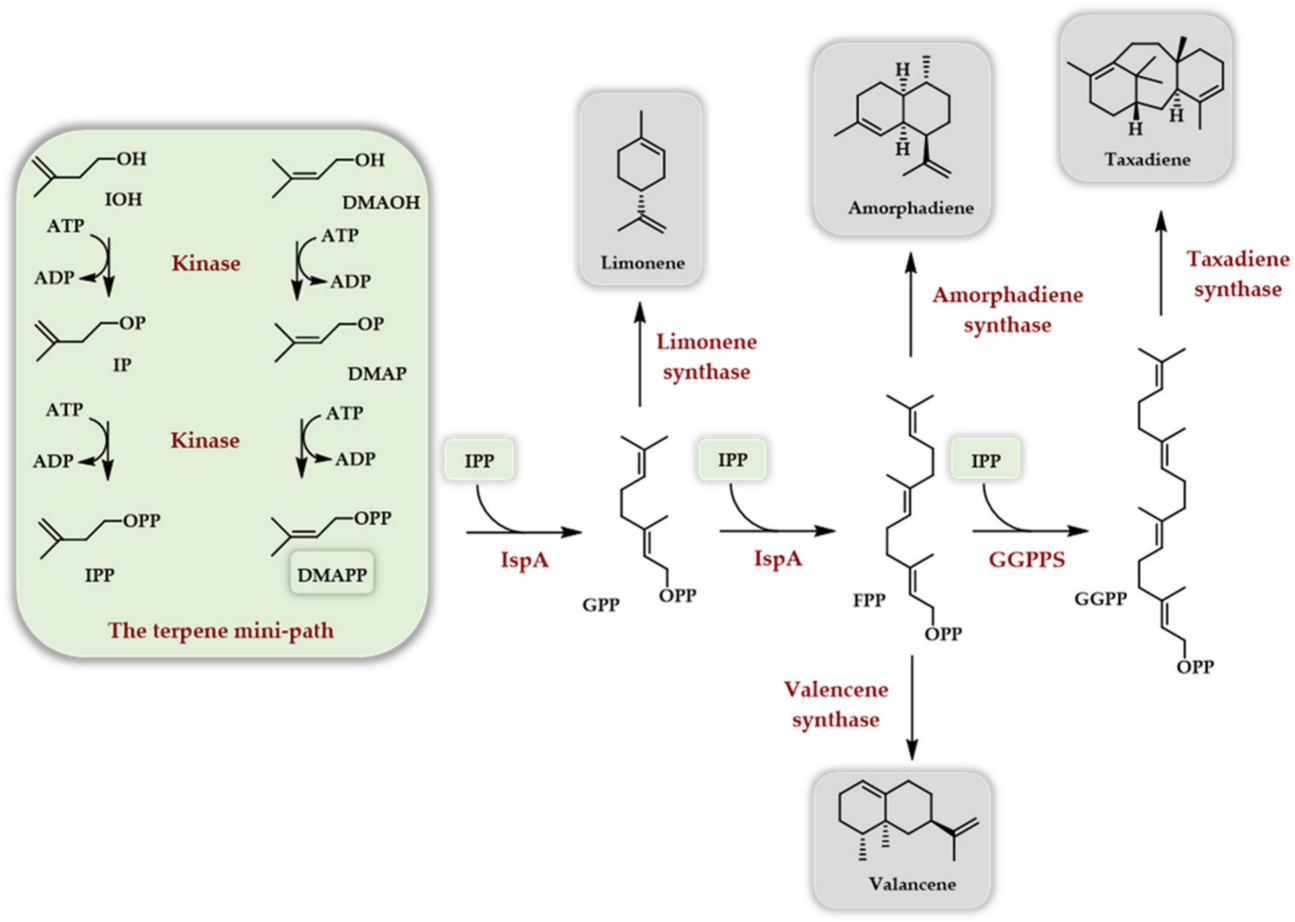
In vitro access to various terpenes thanks to the use of the TMP constitute of the choline kinase from *S. cerevisiae* and the *Arabidopsis thaliana* IPK [31].

Soon after [31], a paper described the use of the *Arabidopsis thaliana* IPK in combination with a true kinase, the choline kinase from *S. cerevisiae*, as the first enzyme of the TMP, called here the IUP. The combination of these two kinases with IDI (isopentenyl diphosphate isomerase), FPPS from *E. coli*, and various terpene synthases (limonene synthase, amorphadiene synthase, and valencene synthase) allowed the in vitro production of the corresponding hydrocarbons (Scheme 6).

Taxadiene synthesis was also attempted using the GGPPS (geranylgeranyl diphosphate synthase) from *Taxus canadensis* and the taxadiene synthase from *Taxus brevifolia*. After the optimization of the various enzyme concentrations, as well as of ATP, DTT, and Mg^2+^ concentrations and other parameters, such as using only IOH with IDI versus a mix of DMAOH and IOH without IDI, a final yield of 220 mg/L in taxadiene was achieved in 9 h with a 65% conversion of added IOH.

**Scheme 7:**
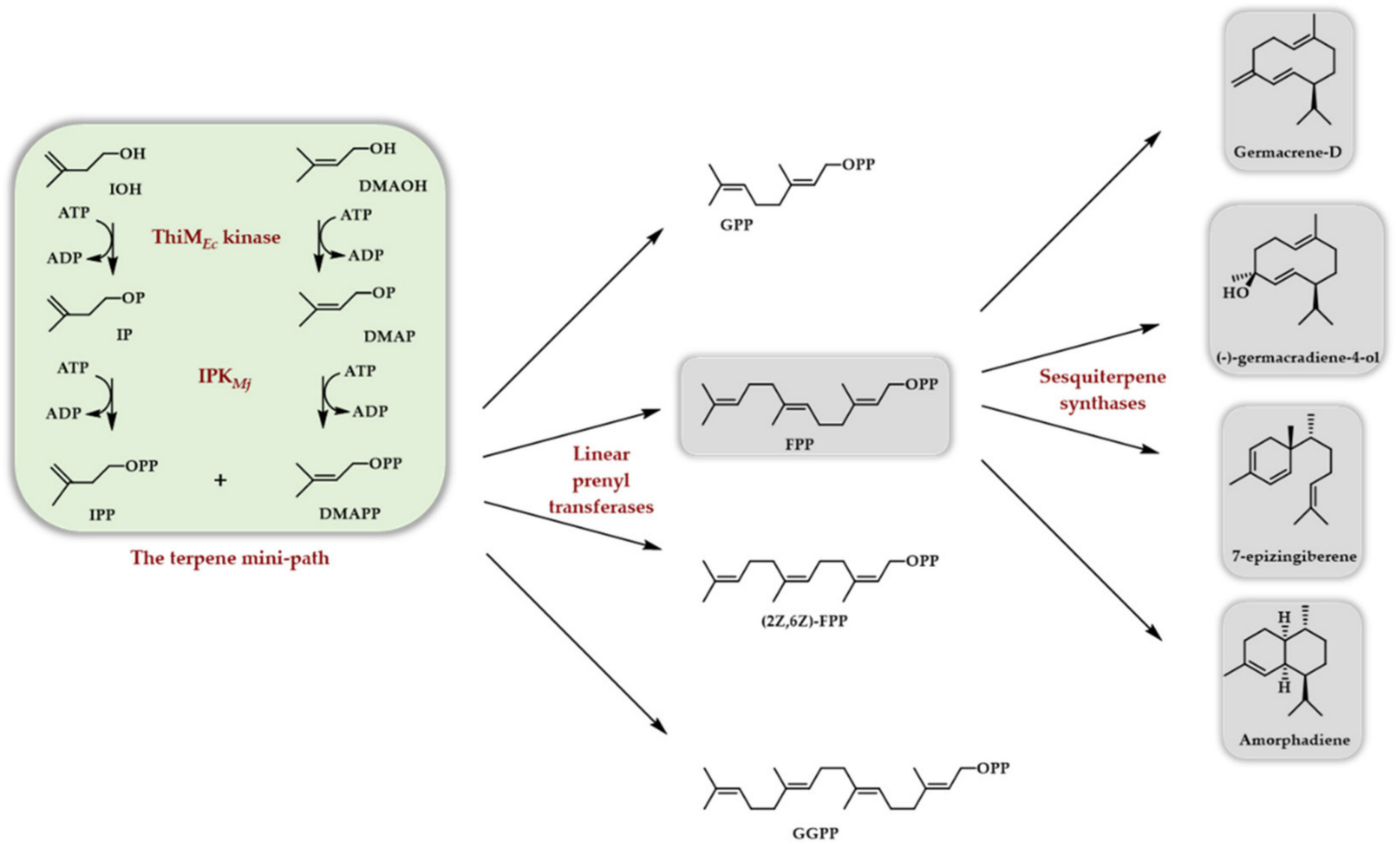
In vitro access to various sesquiterpenes thanks to the use of the TMP constituted of the ThiM kinase from *E. coli* and the *Methanocaldococcus jannaschii* IPK.

In 2020, two papers appeared, both taking advantage of the use of another true kinase (the ThiM kinase from *E. coli*, see above) to phosphorylate DMAOH and IOH into DMAPP and IPP in conjunction with the *Methanocaldococcus jannaschii* IPK [29,30]. In the former case, the formed DMAPP and IPP were used as substrates of different linear prenyl transferases (Scheme 6) to access (*E*,*E*)-FPP, (*Z*,*Z*)-FPP, GPP, and GGPP. Implementing a terpene synthase/cyclase further afforded, depending on the used enzyme, (*S*)-germacrene D, (-)-germacradiene-4-ol, amorpha-4,11-diene, and 7-epi-zingiberene (Scheme 7). The TMP has also been exploited to generate, from homologs of DMAOH and IOH, various linear diphosphates homologous to FPP. Some of them were then used as substrates for germacrene D and amorphadiene sesquiterpene synthases, providing the corresponding unnatural terpenoids [30].

**Scheme 8:**
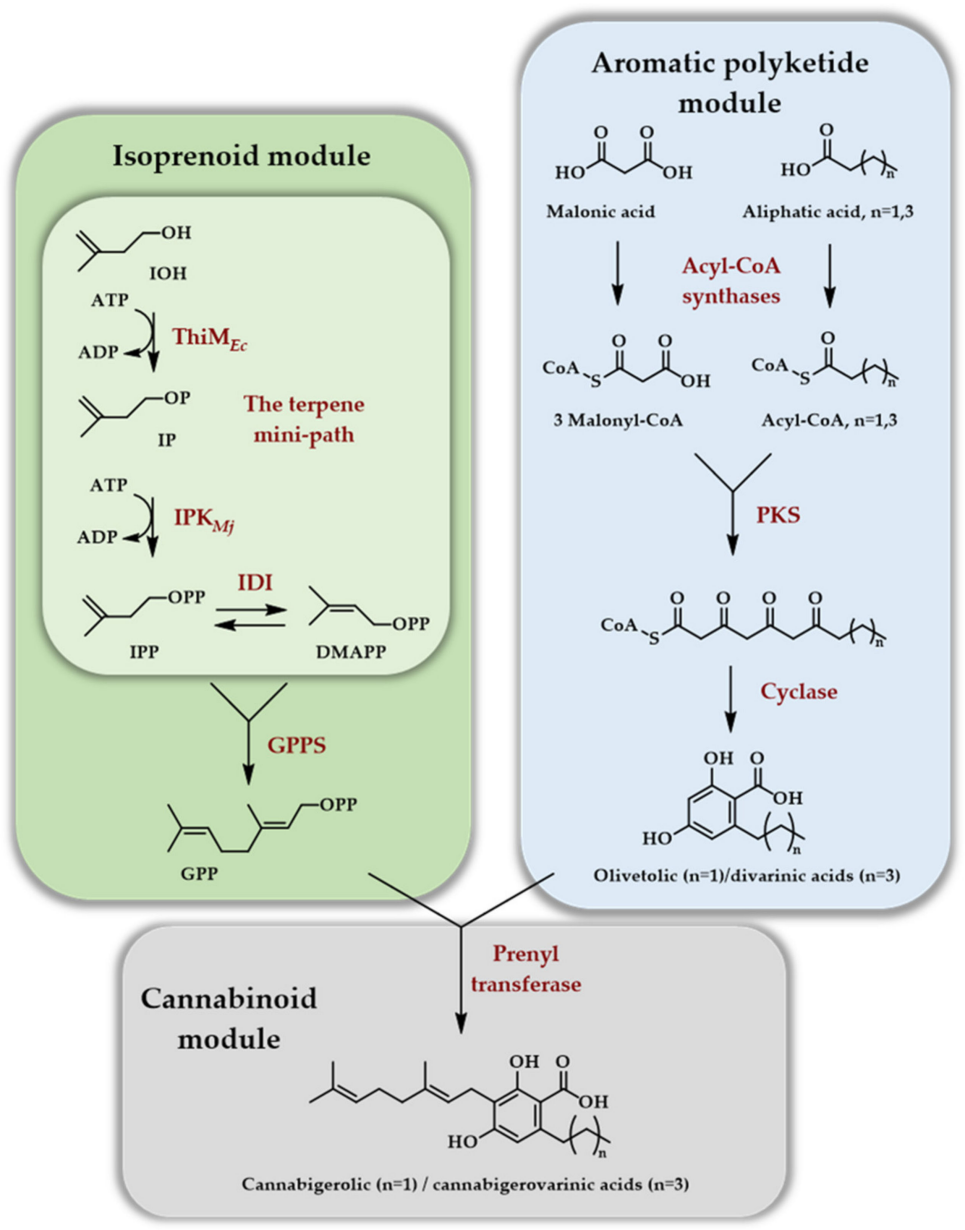
In vitro access to cannabinoids thanks to the TMP involving the ThiM kinase from *E. coli* and the *Methanocaldococcus jannaschii* IPK, as well as an aromatic polyketide module and a cannabinoid module. The ATP regeneration module, as well as ATP consumption for the various modules, except for kinases, are omitted (adapted from [29]).

The second article [29] deals with the generation of cannabinoids, implying many more enzymatic steps than previous reports (12 enzymes in total). This demonstrated that the in vitro enzymatic production of natural products of mixed biosynthetic origin (polyketide/terpene in that case) could be envisioned seriously thanks to the reduced number of enzymes of the TMP. As far as the terpene part is concerned, GPP was produced with a four enzymes cascade starting from IOH using ThiM*_Ec_*, IPK*_Mj_*, IDI*_Ec_*, and FPPS*_Gs_* S82F. The latter, a point mutant of FPPS from *Geobacillus stearothermophilus*, no more catalyzed the formation of FPP but rather of GPP. This so-called ISO module was combined with an ATP regeneration module employing acetyl-phosphate as a sacrificial phosphate donor. The aromatic polyketide module affords either the olivetolic or divarinic acids depending on the length of the hydrocarbon side chain and, when combined with GPP through the cannabinoid module, they afford cannabigerolic acid at 480 mg/L or cannabigerovarinic acid at 580 mg/L titers on a 1 mL scale in 10 h (Scheme 8).

These four examples prove that the TMP offers, compared to the MVA and MEP pathways, the possibility to easily synthesize terpenes and terpenoids in vitro using purified enzymes. Although the enzyme purification is a time-consuming and costly process, the drastic reduction in the number of enzymes needed to access DMAPP and IPP thanks to the TMP (two instead of eighteen) can be exploited to access various terpenoids and could be of interest in the discovery of new terpene synthases as well as in the determination of their substrate promiscuity.

### 4.3. The TMP In Vivo

#### 4.3.1. *Escherichia coli* as Host

**Scheme 9:**
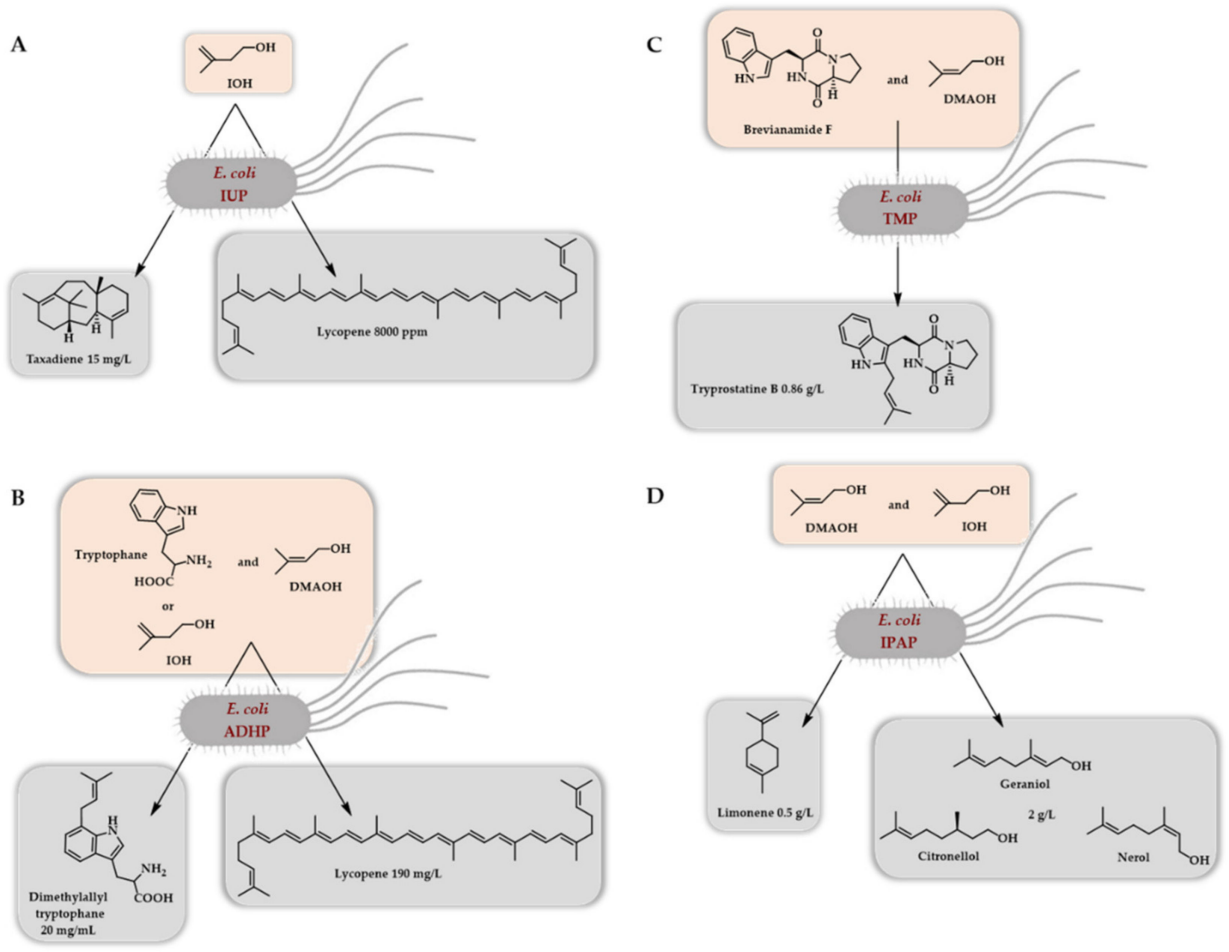
Initial demonstration of the in vivo access to terpenoids (*E. coli*) thanks to the so-called isopentenol utilization pathway (**A**), the alcohol-dependent hemiterpene pathway (**B**), the terpene mini-path (**C**), or the isoprenoid alcohol pathway (**D**) using a two enzymes cascade and starting from DMAOH/IOH to access DMAPP/IPP.

The very first report on a two-step access to DMAPP/IPP (IUP) was mainly dedicated to the in vivo synthesis of lycopene and taxadiene (valencene, limonene, miltiradiene, and amorphadiene synthesis were also attempted) in *E. coli* by the use of a choline kinase from *Saccharomyces cerevisiae* and the *Arabidopsis thaliana* IPK as the two kinases of the artificial pathway [10]. The two corresponding genes were incorporated in an operon in combination with an *idi* gene to balance the DMAPP/IPP ratio. Two plasmids were thus generated, one with a constitutive promoter, the second with an inducible promoter. When combined with a plasmid bearing a lycopene operon, the former outperformed, the best conditions being the use of 25 mM IOH. A further optimization of the plasmid copy number, as well as the promoter strength of the lycopene operon, led to lycopene content of 8000 ppm in a short time (12 h) following induction (Scheme 9A). When taxadiene production was attempted, a 15 mg/L concentration was obtained after 48 h of culture (Scheme 9A). A very interesting result has come from this pioneer work. By analyzing the intracellular concentration of various phosphates and diphosphates (IP, IPP, GPP, FPP, GGPP), it was demonstrated that the flux to IPP using the so-called IUP pathway is larger than that of the native MEP pathway. This also led to the intracellular accumulation of IPP and GGPP, the flux of the IUP being larger than the ones of the downstream lycopene and taxadiene operons.

Soon after this first report, two other papers [11,12] described the access to DMAPP/IPP from DMAOH/IOH using a phosphatase as the first enzyme of the artificial path in combination with an IPK. In one case [11], the genes coding the PhoN phosphatase from *Shigella flexneri* and the *Thermoplasma acidophilum* IPK were cloned into a pETDuet plasmid (forming the so-called alcohol-dependent hemiterpene (ADHP) pathway). This plasmid was then combined in *E. coli* with a plasmid bearing the genes encoding a lycopene biosynthetic path as well as the *idi* gene allowing, at the end of the fermentation, a titer in lycopene of 150 mg/L. Optimization of the concentration of added alcohols (DMAOH and IOH, 5 mM each) further increased the lycopene titer to 190 mg/L. The same genetic construction but replacing the lycopene operon by the gene of a tryptophan prenyl transferase allowed an approximately 20 mg/L production of dimethylallyltryptophan (Scheme 9B). In the other case [12], the V78L mutant of the *Salmonella enterica* phosphatase and the IPK from *Thermoplasma acidophilum* were used in order to transform the chemically synthesized cyclo-dipeptide brevianamide F into the prenylated and cytotoxic derivative tryprostatin B. Based on added brevianamide F and after optimization, a 63% isolated yield in tryprostatin B was obtained after 24 h, at a titer of 860 mg/L (Scheme 9C).

The same year, another report employing the *E. coli* ThiM kinase in combination with *Methanothermobacter thermautotrophicus* IPK, forming the so-called isoprenoid alcohol (IPA) pathway, described the production of various mono-, sesqui-, tri-, and tetraterpenoids [13]. When a truncated version of the geranyl diphosphate synthase from *Abies grandis* was combined with either a geraniol synthase from *Ocinum basilicum* or a limonene synthase from *Mentha spicata*, a mixture of monoterpene alcohols (geraniol, nerol, and citronellol) was obtained at a titer of 2 g/L, as well as limonene at a titer of 0.5 g/L, respectively (Scheme 9D). By using dedicated enzymes, farnesol, diaponeurosporene (a C30 carotenoid), and lycopene were also obtained at titers of 80, 15, and 8 mg/L, respectively.

Recently, the production of linalool was reported using the choline kinase from *Saccharomyces cerevisiae* and the *Arabidopsis thaliana* IPK in combination with a linalool/nerolidol synthase (bLinS) from *Streptomyces clavuligerus* and a GPPS from *Abies grandis* [32]. The optimization of bLinS by mutagenesis to reduce the proportion of formed nerolidol, the use of one or two plasmids to introduce the four coding genes, and the use of different strains of *E. coli* resulted in a linalool titer of 167 mg per liter of organic phase (a 20% *v*/*v* organic layer was used to remove linalool/nerolidol).

#### 4.3.2. Yeasts as Host

**Scheme 10:**
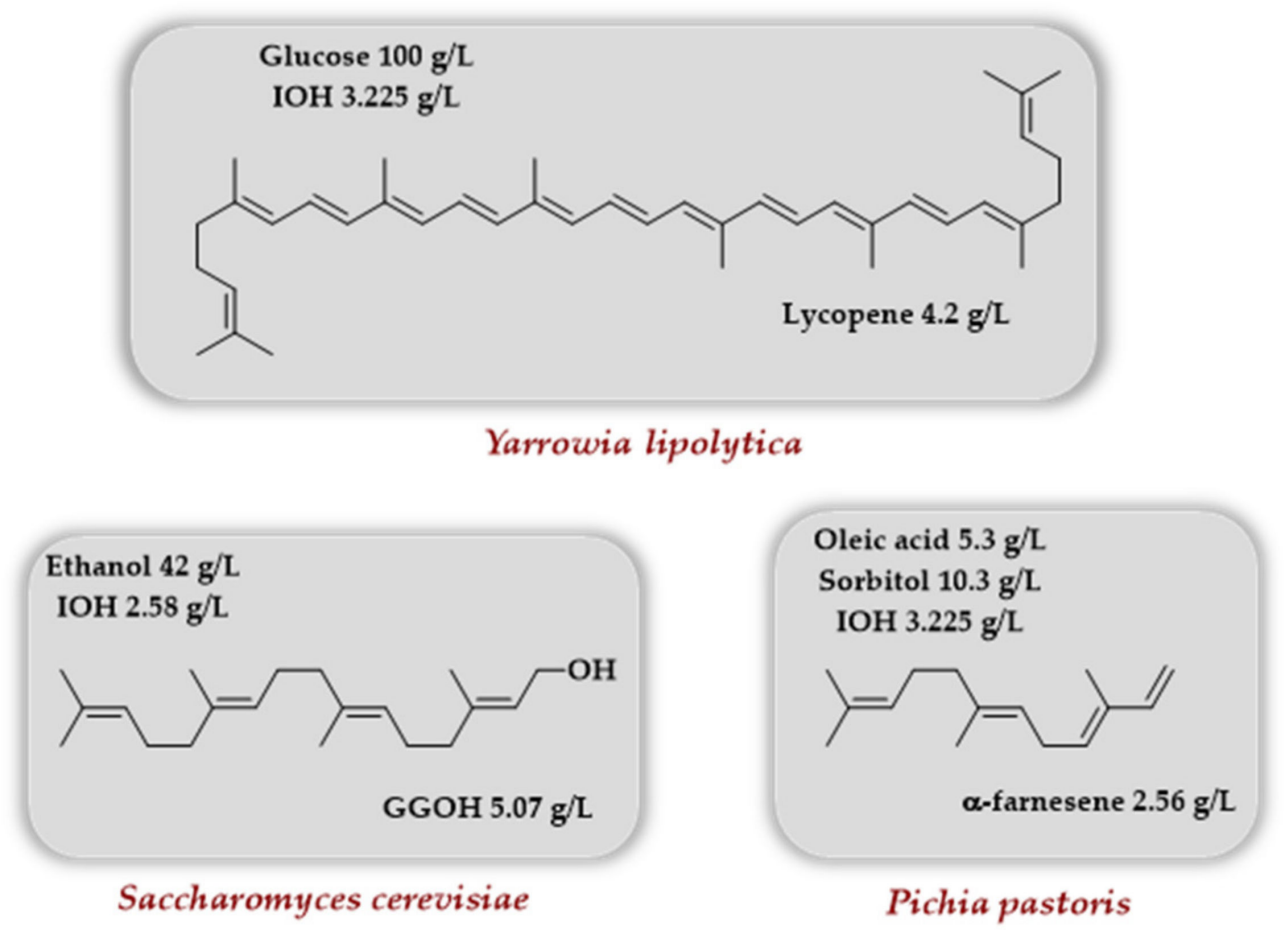
Use of various yeasts to access lycopene, geranylgeraniol, and α-farnesene involving the TMP in conjunction with native or engineered MVA pathway.

Very recently, some reports using the TMP together with an engineered MVA pathway described the production of lycopene, geranylgeraniol, and α-farnesene in various yeasts (Scheme 10). Luo et al. [33] took advantage of the oleaginous nature of the yeast *Yarrowia lipolytica* to produce lycopene with the rationale that the lipid body of this yeast species could store more hydrophobic lycopene than the membranes. It was demonstrated that adding the genes coding for choline kinase and IPK results in enhanced intracellular production of IPP/DMAPP upon IOH addition. When the genes coding for lycopene biosynthesis (*crt*E, *crt*B, and *crt*I from *Lamprocystis purpurea*) as well as the gene coding IDI from *Pseudoescherichia vulgaris* were introduced in *Y. lipolytica* strains, enhanced terpenoid precursors content (GPP, FPP, GGPP), as well as higher lycopene titers, were observed for the strain harboring the TMP upon IOH addition. Enhancement of lycopene titer was further observed when lipid synthesis was promoted either by overexpression of *ACC1* and *DGA1* (coding, respectively, for an acetyl-CoA carboxylase and a diacylglycerol acyltransferase) or by palmitic acid addition to the culture medium (lycopene titers of ~900 and ~600 mg/L, respectively, were obtained). In order to improve the utilization of intracellular FPP and GGPP, the introduction of an additional copy of an *idi* gene and the overexpression of *erg*20, encoding an endogenous FPPS, combined with the addition of palmitic acid in the culture medium, made it possible to reach a lycopene titer of 1.6 g/L in shaken flasks. A final optimization of the palmitic acid concentration (30 mM) and the finding that IOH addition is optimal two days after inoculation led to a final lycopene titer of 4.2 g/L in batch reactor after an 11-day cultivation.

Using *Saccharomyces cerevisiae* as a chassis, the production of geranylgeraniol was studied by Wang et al. [34]. The optimization of an initial strain by overexpression of a truncated version of the 3-hydroxy-3-methylglutaryl-coenzyme A reductase gene (*tHMG1*), the use of a GGPPS-FPPS fusion protein, the use of a dodecane layer, and the deletion of the gene *ROX1* (transcriptional repressor of the sterol pathway) allowed, after 7 days, a final GGOH titer of 3.66 g/L in a fed-batch cultivation. The introduction of the TMP by overexpression of the *Arabidopsis thaliana ipk* (the choline kinase being endogenous), as well as the addition of 30 mM IOH after 2 days, improves the final GGOH concentration to 4.56 g/L (24.6% increase), the latter being further increased to 5.07 g/L upon continuous addition of ethanol (0.35 g/L/h) after day 4.

Liu et al. used the yeast *Pichia pastoris* to target the TMP to the peroxisome and produce α-farnesene [35]. First, the α-farnesene synthase (αFS) from *Malus domestica* was selected from a panel of 20 αFSs as the enzyme producing the highest concentration in α-farnesene when introduced in *P. pastoris* cytoplasm (0.44 g/L). A first optimization of the MVA pathway in the cytoplasm (overexpression of *tHMG1*, *idi1* and *erg20*) led to a 0.67 g/L titer in α-farnesene. A further increase to 1.01 g/L was noted when *EcPDH*, the gene coding the pyruvate dehydrogenase from *E. coli*, was overexpressed to increase the acetyl-CoA supply. In comparison, when the gene coding the αFS from *Malus domestica* was targeted to the peroxisome, a 0.53 g/L titer in α-farnesene was observed, increased to 1.32 g/L when the TMP (with IOH addition) was also targeted to the peroxisome as well as *idi1* and *erg20*. With an extra copy of these two genes, a further increase to 1.69 g/L was observed. Combining both optimizations (cytoplasm + peroxisome) gave a 2.18 g/L titer, and the highest titer of 2.56 g/L in 72 h was attained in shake flasks with carbon source cofeeding (oleic acid, sorbitol).

## 5. Discussion and Perspectives

This review summarized the recent development of the artificial TMP to (bio)synthesize terpenoids starting from DMAOH and IOH. Numerous advantages are associated with such a reduced pathway. The first and most obvious one is the drastic reduction in the number of enzymes needed to access the two universal precursors, DMAPP and IPP, from 18 for the MVA and MEP pathways to only two (or three if IDI is considered) in the TMP. The limited number of enzymes to deal with offers the opportunity to construct more easily in vitro enzymatic cascades to access terpenoids. The successful productions of a prenylated diketopiperazine, tryprostatin B at a titer of 3 g/L, of a precursor of taxol, taxadiene at 220 mg/L, and of cannabigerolic acid at 480 mg/L or cannabigerovarinic acid at 580 mg/L are great achievements in the field. Another important point to be raised is the fact that the TMP only needs ATP as a cofactor and no reducing cofactors to the contrary of the MVA and MEP pathways, hence simplifying the buildup of the cascade. Furthermore, DMAOH and IOH are cheap industrial compounds, available in bulk and partially soluble in water, all important things from both an environmental and an industrial point of view. Another interesting point with the use of the TMP is its ability to easily access, for the first time, non-natural terpenoids by using analogs of DMAOH or IOH or of both [30]. Indeed, the two enzymes of the TMP used by Johnson et al. have been shown to be promiscuous enough to generate modified DMAPPs and IPPs, which can subsequently be incorporated into different terpenoids [30]. All these points are also beneficial when considering the in vivo use of the TMP. Indeed, only two genes are needed to construct the TMP, their introduction being easily completed in the chassis of choice through plasmid transformation. The only need for the TMP in vivo is the supply of ATP, something provided by the microorganism used as a chassis. As DMAOH and IOH are small, neutral, and rather hydrophobic chemicals, they easily pass the biological membranes and, thus, can be internalized without the need for a transporter. Probably, the main in vivo advantage of the TMP is its total decoupling (except for ATP production) from the central metabolism, with no regulation being associated with the use of the two enzymes. The TMP can thus be optimized on its own to adjust the flow of DMAPP/IPP on demand. Another important point is that the DMAOH/IOH ratio to be added to the culture medium can be adjusted at will using different ratios suitable for the production of monoterpenes (theoretical ratio 1/1), sesquiterpenes (theoretical ratio 1/2), diterpenes (theoretical ratio 1/3), triterpenes (theoretical ratio 1/5), and tetraterpenes (theoretical ratio 1/7). Another option in this context is the use of an IDI-like enzyme to interconvert DMAPP to IPP and vice versa. The addition of DMAOH/IOH can also be done at will in the case of the TMP, particularly after induction of the latter, which is not possible with the MVA and MEP pathways where the source of carbon and energy (glucose, glycerois also the source of biosynthetic carbon. Some of these advantages have been exploited to in vivo access lycopene (190 mg/L), tryprostatin B (860 mg/L), limonene (500 mg/L), and geranoids (2 g/L) using *E. coli* as a chassis. Higher yields were seen when a yeast chassis was used to produce terpenoids, but, in all the reported cases, both the TMP and the MVA pathways were used together to produce different terpenoids, such as lycopene (*Yarrowia lipolytica*, 4.2 g/L, 11 days), geranylgeraniol (*Saccharomyces cerevisiae*, 5.07 g/L, 7 days), and α-farnesene (*Pichia pastoris*, 2.56 g/L, 3 days).

Besides all the advantages described above, some disadvantages can be drawn when it comes to the use of the TMP either in vitro or in vivo. The first point is that DMAOH and IOH are, for now, chemically synthesized starting from isobutene and formaldehyde through the Prins reaction. Therefore, they cannot be used to access terpenoids with a natural label. Nevertheless, isobutene and formaldehyde can potentially be bio-sourced, the former being produced through fermentation [36] and the latter by oxidation of bio-methanol. Another negative point of the use of DMAOH/IOH is that they are toxic to cells and limit growth beyond a threshold depending on the microbial chassis used. Thus, the time of addition and the rate of addition of the two alcohols are probably to be carefully controlled in in vivo experiments.

The use of the TMP either in vitro or in vivo and either alone or in combination with the MVA or the MEP pathways holds great promise both at the bench and industrial scales. In fact, the ease of construction either in the form of an enzymatic cascade or within a microbial chassis makes the TMP particularly attractive. Numerous improvements could be envisioned either in vitro and/or in vivo to further enhance its attractiveness. In the former case, introduction of a low-cost ATP regeneration system will be beneficial; indeed, using PEP as a sacrificial phosphate donor is far from optimal in terms of costs. Acetylphosphate has already been used efficiently as a phosphate donor in conjunction with acetate kinase [29], but other cheaper systems, such as the polyphosphate/polyphosphate kinase couple, can also be envisioned. The industrial use of isolated enzymes is usually associated with their immobilization due to the extended shelf life and adoption of a continuous process leading to large increases in productivity. The two-enzyme nature of TMP is, therefore, particularly interesting in this context. The key in the development of the TMP has been the discovery of promiscuous enzymes capable of catalyzing the monophosphorylation of DMAOH and/or IOH allowing, when combined with IPKs, to generate DMAPP/IPP. Various phosphatases, as well as choline kinase, ThiM kinase, and even IPK, were tested and used in the framework of the TMP. In our experience, the kinase activity of the first TMP enzyme leading from DMAOH/IOH to DMAP/IP is lower than that of the IPKs used in the second phosphorylation step and leading to DMAPP/IPP. A search in the biodiversity or protein engineering of the already known C5-alcohol kinase could be key in the development of an improved TMP, leading to a balanced activity of the two kinases. Another point of improvement could be the balancing of the kinase activities of the two TMP enzymes towards DMAOH and IOH, as well as DMAP and IP. Indeed, depending on the terpenoid of interest, one or more IPP is needed as an extender unit for only one DMAPP acting as a starter unit. Thus, the rate of synthesis of the DMAPP will probably have to be adapted to that of the IPP for each type of terpenoid produced (mono-, sesqui-, di-, tri, tetraterpenes) to avoid the accumulation of toxic diphosphate compounds in the cell. Finally, from the results analyzed in this review, the use of yeast eukaryotic cells seems to be preferable to prokaryotic ones since higher final terpenoid titers were observed (multi grams versus multi hundreds of mgs). It should, nevertheless, be stressed that, in the three reported cases in yeast, the MVA plays a major role in terpenoid production, the TMP acting as a complement. There is, thus, probably a great deal of room to improve the terpenoid production both in prokaryotic and eukaryotic cells by using the TMP.
